# Comparison of Clinical Symptoms and Cardiac Lesions in Children with Typical and Atypical Kawasaki Disease

**DOI:** 10.3390/medsci7040063

**Published:** 2019-04-18

**Authors:** Maryam Behmadi, Behzad Alizadeh, Abdolreza Malek

**Affiliations:** 1 Department of Pediatric Diseases, Faculty of Medicine, Mashhad University of Medical Sciences, Mashhad 99191-91778, Iran; behmadim941@mums.ac.ir; 2 Pediatric and Congenital Cardiology Division, Department of Pediatric Diseases, Faculty of Medicine, Mashhad University of Medical Sciences, Mashhad 99191-91778, Iran; alizadehb@mums.ac.ir

**Keywords:** Kawasaki disease, typical, atypical, cardiac lesions

## Abstract

The present study was performed to evaluate the clinical symptoms and cardiovascular complications in patients with typical and atypical Kawasaki disease (KD). This retrospective study was conducted on the medical records of 176 patients with KD for three years. The study population was divided into two groups of typical and atypical based on the KD clinical criteria. The two groups were compared in terms of demographic data, clinical symptoms, cardiac lesions, and laboratory markers. Based on the diagnostic criteria, 105 (60%) and 71 (40%) patients were diagnosed with typical and atypical KD, respectively. The mean age of the typical patients (38.16 months) was higher than that of the atypical group (24.03 months) at the time of diagnosis (*p* < 0.05). The results revealed no significant difference between the two groups regarding the seasonal distribution of KD onset (*p* = 0.422). However, the most common season for the diagnosis of the disease was spring, followed by winter. There was no significant difference between the two groups in terms of fever duration (*p* = 0.39). Furthermore, vomiting was more common in the atypical patients than in the typical group (*p* = 0.017). In terms of the cardiac lesions, ectasia (*p* = 0.005) and lack of tapering of the distal coronary vessels (*p* = 0.015) were more frequently detected in the atypical group than in the typical group. Considering the laboratory findings, thrombocytosis (*p* = 0.010) and anemia (*p* = 0.048) were more common in the atypical group, compared to those in the typical group. On the other hand, the typical group had a higher serum alanine aminotransferase level (adjusted for age) (*p* = 0.012) and Hyponatremia (serum sodium concentration ≤130 mmol/L) (*p* = 0.034). Based on the findings of the current study, the fever duration from onset to diagnosis was slightly more in atypical KD patients than in the typical group, but not statistically significant, possibly due to more timely diagnosis of atypical KD. There was no difference in coronary aneurysm between the two groups at the time of diagnosis. The atypical group had a higher frequency of coronary ectasia and lack of tapering, indicating cardiac involvement. Consequently, these conditions should be given more attention in the atypical patients. Furthermore, the higher frequency of anemia and thrombocytosis in the atypical patients can be useful for diagnosis of this kind of KD.

## 1. Introduction

Kawasaki disease (KD) was first reported in Japan in 1967 by Tomisaku Kawasaki observing 50 patients [1]. This disease is one of the most common vasculitis conditions in childhood [2]. The exact etiology of KD still remains unknown [3]. A combination of environmental and genetic factors accounts for the incidence of KD. Accordingly, it has a higher incidence rate in specific seasons and Asian populations [4].

KD patients are divided into two groups of typical and atypical. The five principal clinical features of KD are polymorphic rash, bilateral nonexudative bulbar conjunctivitis, oral changes (e.g., red and cracked lips, strawberry tongue, erythema of the oropharynx), extremity changes (e.g., swollen hands and feet, erythema of the palms and soles, and/or periungual desquamation in two weeks), and cervical lymphadenopathy (≥1.5 cm, usually unilateral) [5].

The diagnosis of typical KD is based on the presence of four out of the five principal clinical criteria, accompanied by at least five days of fever. Furthermore, atypical KD is characterized by a fever for a minimum of five days and the presence of two or three of the above criteria. The diagnosis of atypical KD is based on echocardiographic findings indicating the involvement of the coronary arteries [5,6,7] and the laboratory markers. McCrindle et al. [7] suggested diagnostic pathways for suspected atypical patients with respect to the laboratory findings (white blood cell, WBC) count of ≥15,000 mm^3^, platelet count of ≥450,000 mm^3^, pyuria of ≥10 WBC/high-power field (HPF), albumin level of ≤3 g/dL, elevation of alanine aminotransferase (ALT) and anemia for age). More than three laboratory criteria support the diagnosis of atypical KD [8]. Delayed diagnosis and treatment of atypical KD can lead to an increased risk of cardiac complications [9].

KD is the leading cause of acquired heart disease in children [10]. This disease is associated with the development of myocarditis and coronary artery abnormalities [11]. The main features of coronary artery involvement in KD are perivascular brightness, aneurysm formation, ectasia, dilatation, and lack of tapering of the distal coronary vessel [12]. Coronary artery aneurysms are defined as local dilatations in the coronary artery that are 1.5 times greater than normal adjacent segments. Coronary artery ectasia is described as diffuse arterial dilatations that involve 50% or more of the length of the artery [13,14].

To the best of our knowledge, no study in Iran has investigated KD comparing typical and atypical patients. With this in mind, the present study was conducted to evaluate the clinical symptoms and cardiovascular complications in typical and atypical KD patients.

## 2. Materials and Methods

This retrospective study was conducted on the medical records of 194 KD patients admitted to the rheumatology departments of two hospitals, namely Akbar Pediatric Hospital and Imam Reza Hospital, in Mashhad, Iran, between 21 March 2015 and 21 March 2018. Mashhad is the second most populous city in Iran. It has a population size of 3,001,184 (based on the 2016 census). This study was approved by the Ethics Committee of Mashhad University of Medical Sciences, Mashhad, Iran (ethical code: IR.MUMS.fm.REC.1396.562).

Eighteen patients were excluded from the study due to their incomplete medical records. The patients were divided into two groups of typical and atypical based on the KD clinical criteria. The demographic data, clinical signs, echocardiographic findings, and laboratory markers were recorded for each patient in a standardized form. It must be specified that these data were obtained at the acute phase of KD (i.e., the first 10 days of the disease).

The data recorded for each patient included the fever duration, gender, seasonal onset, clinical signs (e.g., polymorphic rash, bilateral nonexudative bulbar conjunctivitis, oral changes, extremity changes, and cervical lymphadenopathy), nonspecific symptoms (e.g., arthralgia, diarrhea, and vomiting), cardiac lesions (e.g., myocarditis, pericardial effusion, coronary artery abnormalities (perivascular brightness, aneurysm formation, ectasia, dilatation, and lack of tapering of the distal coronary vessel), valvular lesions, myocardial infarction) and laboratory markers.

In some studies, Z score was used to grade the severity of coronary artery involvement. However, with respect to the other studies, we used criteria for cardiac lesions in Kawasaki disease as defined by the Japanese Ministry of Health [1]. Coronary dilatation was defined as a maximum internal lumen diameter of >3 mm in children aged younger than five years or >4 mm in children aged five years or older. The aneurysms are classified as small (<5 mm internal diameter), medium (5–8 mm internal diameter), or giant (>8 mm internal diameter) [15]. The diagnosis of myocarditis in KD patients was according to tachycardia in physical examination and a low ejection fraction (EF) in echocardiography. In our study, valvular lesions refer to mitral or aortic regurgitation (from mild to severe).

The laboratory markers included a white blood cell (WBC) count of ≥15,000 mm^3^, erythrocyte sedimentation rate (ESR) of ≥40 mm/h, C-reactive protein (CRP) level of ≥30 mg/L, platelet count of ≥ 450,000 mm^3^, pyuria of ≥10 WBC/HPF, albumin level of ≤3 g/dL, and sodium concentration of ≤130 mmol/L. The ALT and hemoglobin levels were adjusted for age.

The data were analyzed by Mann–Whitney, Chi-square, and Fisher’s exact tests. *p*-values of less than 0.05 were considered statistically significant.

## 3. Results

During the study period, 194 KD patients were admitted to the two hospitals under investigation; however, 176 medical records were included in the study since the rest were incomplete. Regarding the type of KD, 105 (60%) and 71 (40%) patients were diagnosed with typical and atypical KD, respectively. Table 1 presents the demographic data of two groups. Based on the results of the Mann–Whitney test, the mean age of the typical patients (38.16 months) was higher than that of the atypical group (24.03 months) at the time of diagnosis (*p* < 0.05).

In terms of gender distribution, 63.6% of the KD patients were male, resulting in a male to female ratio of 1.75/1. However, the results of the Chi-square test revealed no significant difference between the two groups in terms of male predilection (*p* = 0.561). Regarding the season of the KD onset, 33.1%, 20.6%, 20%, and 26.3% of the cases had occurred in spring, summer, autumn, and winter, respectively. The most common season of diagnosis was spring, followed by winter. According to the results of the Chi-square test, there was no significant difference between the two groups in terms of the seasonal distribution of KD onset (*p* = 0.422).

Table 2 demonstrates the clinical characteristics of the typical and atypical KD patients. All the patients in both groups had fever. Fever duration from onset to diagnosis was 7.33 ± 3.45 days and 7.73 ± 3.42 days in typical and atypical groups, respectively. There was no significant difference between the two groups in terms of fever duration (*p* = 0.39). Among the five principal criteria of KD, oral changes, conjunctivitis, and rash respectively had the highest frequency in the typical patients. Regarding the atypical group, conjunctivitis, oral changes, and rash were found to have the highest frequency.

The results of the Chi-square test revealed no significant difference between the typical and atypical patients regarding the frequency of arthralgia and diarrhea as nonspecific symptoms (*p* = 0.657 and *p* = 0.055, respectively). However, our results showed that vomiting was more common in the atypical patients than in the typical group (*p* = 0.017). It must be noted that only seven KD patients had gallbladder hydrops, the frequency of which was not significantly different between the two groups (*p* = 0.703).

Table 3 lists the frequency of cardiac lesions in the acute phase among the typical and atypical KD patients. The results of our study demonstrated that 59% of the KD patients had at least one of the cardiac lesions. The frequencies of these lesions were obtained as 58% and 61% in the typical and atypical groups, respectively. There was a significant difference between the typical and atypical patients in terms of ectasia and lack of tapering of the distal coronary vessel. Based on the results of Fisher’s exact test, ectasia (*p* = 0.005) and lack of tapering of the distal coronary vessel (*p* = 0.015) were more frequent in the atypical group than in the typical group.

Our data analysis also showed that there was no significant difference between the two groups in terms of aneurysm (*p* = 0.354). Among 11 patients with aneurysm, only one patient had a giant aneurysm and the other patients had small to medium size aneurysms.

As the results of the Chi-square test showed, the two groups were not significantly different in terms of myocarditis (*p* = 0.990), valvular lesions (*p* = 0.060), pericardial effusion (*p* = 0.145), dilatation (*p* = 0.229), and perivascular brightness (*p* = 0.147).

In our study, valvular lesions refer to mitral or aortic regurgitation (from mild to severe). The frequency of mitral regurgitation was 97.5% (39 of 40). Mitral insufficiency was mild in 36 and moderate in three patients. The only case of aortic regurgitation was found in the atypical group.

Table 4 tabulates the laboratory findings in the patients with typical and atypical KD. The Chi-square test revealed no significant difference between the typical and atypical groups in terms of ESR (*p* = 0.685), CRP (*p* = 0.804), WBC (*p* = 0.617), and albumin levels (*p* = 0.987).

The results of the Chi-square test showed that thrombocytosis (*p* = 0.010) and anemia (*p* = 0.048) were more commonly observed in the atypical group than in the typical group. However, the typical group had a higher ALT level (*p* = 0.012) and Hyponatremia (*p* = 0.034), compared to the atypical group. According to laboratory findings, in the acute phase only five patients (2 typical and 3 atypical) had thrombocytopaenia (platelet counts (PLT) ≤150,000 mm^3^).

## 4. Discussion

The results of our research showed that the atypical patients had a lower mean age than the typical patients. This finding is consistent with the results of previous studies, demonstrating that atypical patients were younger than typical patients [8,16,17]. Based on the evidence, KD more commonly affects boys than girls [18,19]. Our results demonstrated a higher KD prevalence in males as compared to that in females (male:female ratio = 1.75/1). However, there was no significant difference between the typical and atypical patients in terms of gender distribution.

Based on the findings of the present study, most of the KD cases occurred in spring and winter. Likewise, spring and winter have been reported as the seasonal peaks for KD in a number of studies. The predominant season for KD occurrence varies across different countries [20]. The seasonal variation in the incidence of KD is related to the changes in tropospheric wind patterns [4,21,22]; however, this approach cannot be applied for every country.

In the current study, oral changes, followed by conjunctivitis and rash, had the highest frequency in the patients with typical KD. In the atypical group, conjunctivitis, oral changes, and rash were the clinical manifestations with the highest frequency. Manlhiot et al. [23] and Gorczyca et al. [24] reported conjunctivitis, oral changes, and rash as the more frequent presentations of KD in both groups. Furthermore, Perrin et al. [25] demonstrated a higher frequency of these three clinical manifestations in atypical patients. They also reported a higher frequency of oral changes, changes in the extremities, rash, and conjunctivitis in the typical group.

Giannouli et al. [26] found that conjunctivitis, oral changes, and rash were more frequent in the typical group. Their results also showed that oral changes, rash, and lymphadenopathy were more common in the atypical patients. Maric et al. [27] observed that oral changes, rash, and conjunctivitis were more common in the typical patients. Their results revealed a higher frequency of conjunctivitis, rash, and oral changes in the atypical group.

In terms of the nonspecific symptoms, there was no significant difference between the typical and atypical patients regarding arthralgia and diarrhea. However, in the present study, vomiting was more commonly observed in the atypical patients than in the typical group.

Cardiac lesions are the serious complications of KD. In this study, cardiac lesions such as myocarditis, pericardial effusion, coronary artery abnormalities (e.g., perivascular brightness, aneurysm formation, ectasia, dilatation, and lack of tapering of the distal coronary vessel), valvular lesions, and myocardial infarction were investigated in the typical and atypical patients. The results revealed that 59% of the KD patients had at least one of the cardiac lesions. The frequency distributions of these lesions were 58% and 61% in the typical and atypical groups, respectively.

There was a significant difference between the typical and atypical patients in terms of the frequency of ectasia and lack of tapering of the distal coronary vessel. In this regard, these two lesions were more frequent in the atypical group than in the typical patients. Nonetheless, there was no significant difference between the two groups regarding the other cardiac lesions, especially aneurysm. There are a limited number of studies focusing on the comparison of cardiac lesions between typical and atypical patients. In a study performed by Ha et al. [28], no significant difference was observed between typical and atypical patients in terms of coronary artery aneurysm. However, they reported a significant difference in dilatation between the two groups.

Shilvalingham et al. [29] investigated the clinical presentations and long-term outcomes of atypical patients and compared them with those of typical patients. They found that short- and long-term coronary outcomes were similar in both groups. In another study carried out by Ozdemir et al. [30], coronary artery abnormality had a similar frequency in typical and atypical KD patients. Maric et al. [27] found that the development of coronary artery aneurysm in atypical KD patients was more common, compared to that in typical patients.

The WBC, ESR, and CRP levels are widely applied as the indices of the severity of inflammation. The results of the current study showed no significant difference between the typical and atypical groups in terms of ESR, CRP, and WBC levels. Similarly, Perrin et al. [25] found no significant difference between the typical and atypical patients in terms of ESR and CRP levels. In another study conducted by Maric et al. [27], no significant difference was reported in ESR and CRP levels between the two groups.

Anemia is a common clinical feature in KD patients [31]. In the current study, anemia was more common in the atypical patients than in the typical group. Ha et al. [28] found that atypical patients had a lower level of hemoglobin compared to the typical group. Our results also showed that thrombocytosis (i.e., platelet count of ≥450,000) was more frequent in the atypical group than in the typical group. Likewise, in the study by Ha et al. [28], the atypical group had a higher platelet count than the typical group. Nonetheless, Manlhiot et al. [23] detected no significant difference in platelet count between the typical and atypical groups.

According to our results, the ALT level was higher in the typical group than in the atypical group, which is in line with the results reported by Maric et al. [27] and Perrin et al. [25]. Regarding the other laboratory markers, the typical and atypical groups showed no significant difference in terms of serum albumin and pyuria. However, hyponatremia (serum sodium ≤130 mmol/L) was significantly more common in the typical group than in the atypical group.

## 5. Conclusions

As the findings of the present study indicate, the fever duration from onset to diagnosis was slightly more in atypical KD patients than in the typical group, but not statistically significant, possibly due to more timely diagnosis of atypical KD. There was no difference in coronary aneurysm between the two groups at the time of diagnosis.

Ectasia and lack of tapering were more common in the atypical group than in the typical group. Regarding this, cardiac involvement should be given special attention in the atypical patients. Furthermore, the higher frequency of anemia and thrombocytosis in atypical patients can be useful in the detection of this kind of KD.

## Figures and Tables

**Table 1:** Demographic information of typical and atypical Kawasaki disease (KD) patients.

Parameter	Whole Group *n* = 176 *n* (%)	Typical Group *n* = 105 *n* (%)	Atypical Group *n* = 71 *n* (%)	*p*-Value
**Gender**				
Male	112 (63.6%)	65 (61.9%)	47 (66.2%)	0.561
Female	64 (36.4%)	40 (38.1%)	24 (33.8%)	
Age (Month)	32.43 (2–114)	38.16 (3–114)	24.03 (2–108)	**<0.001**
**Seasonal Onset**				
Spring	58 (33.1%)	37 (35.6%)	21 (29.6%)	0.422
Sumer	36 (20.6%)	18 (17.3%)	18 (25.4%)	
Autumn	35 (20%)	19 (18.3%)	16 (22.5%)	
Winter	46 (26.3%)	30 (28.8%)	16 (22.5%)	

Statistical significance shown in bold.

**Table 2:** Clinical characteristics in typical and atypical KD patients.

Parameter	Whole Group *n* = 176 *n* (%)	Typical Group *n* = 105 *n* (%)	Atypical Group *n* = 71 *n* (%)	*p*-Value
**Clinical Signs**				
Polymorphic rash	124 (70.5%)	92 (87.6%)	32 (45.1%)	**<0.001**
Extremity changes	82 (46.6%)	71 (67.6%)	11 (15.5%)	**<0.001**
Bulbar conjunctivitis	145 (82.4%)	101 (96.2%)	44 (62%)	**<0.001**
Oral changes	143 (81.3%)	103 (98.1%)	40 (56.3%)	**<0.001**
Cervical lymphadenopathy	81 (46%)	67 (63.8%)	14 (19.7%)	**<0.001**
**Nonspecific Symptoms**				
Diarrhea	22 (12.5%)	9 (8.6%)	13 (18.3%)	0.055
Vomiting	20 (11.4%)	7 (6.7%)	13 (18.3%)	**0.017**
Arthralgia	13 (7.4%)	7 (6.7%)	6 (8.5%)	0.657
Gallbladder hydrops	7 (4%)	5 (4.8%)	2 (2.8%)	0.703

Statistical significance shown in bold.

**Table 3:** Cardiac lesions in the acute phase in typical and atypical KD patients.

Parameter	Whole Group *n* = 176 *n* (%)	Typical Group *n* = 105 *n* (%)	Atypical Group *n* = 71 *n* (%)	*p*-Value
**Myocarditis**	7 (4%)	4 (3.8%)	3 (4.2%)	0.990
**Valvular lesions**	40 (22.7%)	29 (67.6%)	11 (15.5%)	0.060
**Pericardial effusion**	8 (4.5%)	7 (6.7%)	1 (1.4%)	0.145
**Ectasia**	24 (13.6%)	8 (7.6%)	16 (22.5%)	**0.005**
**Dilatation**	12 (6.8%)	5 (4.8%)	7 (9.9%)	0.229
**Aneurysm**	11 (6.3%)	5 (4.8%)	6 (8.5%)	0.354
**Perivascular brightness**	49 (27.8%)	25 (23.8%)	24 (33.8%)	0.147
**Lack of tapering**	12 (6.8%)	3 (2.9%)	9 (12.7%)	**0.015**

Statistical significance shown in bold.

**Table 4:** Laboratory findings at diagnosis in typical and atypical KD patients.

Parameter	Whole Group *n* = 176 *n* (%)	Typical Group *n* = 105 *n* (%)	Atypical Group *n* = 71 *n* (%)	*p*-Value
CRP ≥ 30 mg/L	141 (84.4%)	85 (85%)	56 (83.6%)	0.804
ESR ≥ 40 mm/h	166 (96.5%)	100 (97.1%)	66 (95.7%)	0.685
PLT ≥ 450000 mm^3^	84 (49.7%)	42 (41.6%)	42 (61.8%)	**0.010**
Sodium ≤ 130 mmol/L	31 (19.6%)	24 (25%)	7 (11.3%)	**0.034**
Pyuria ≥ 10 WBC/HPF	21 (16.5%)	12 (14.8%)	9 (19.6%)	0.489
Albumin ≤ 3 g/dL	8 (5.8%)	5 (5.8%)	3 (4.4%)	0.987
Hemoglobin adjusted for age	122 (72.6%)	67 (67%)	55 (80.9%)	**0.048**
ALT (U/L) adjusted for age	49 (30.4%)	37 (37.8%)	12 (19%)	**0.012**
WBC ≥ 15000 mm^3^	66 (39.1%)	41 (40.6%)	25 (36.8%)	0.617

CRP—C-reactive protein; ESR—erythrocyte sedimentation rate; PLT—platelet counts; WBC—white blood cell; ALT—alanine aminotransferase. Statistical significance shown in bold.
